# Inhibition of Bacterial Virulence: Drug-Like Molecules Targeting the *Salmonella enterica* PhoP Response Regulator

**DOI:** 10.1111/j.1747-0285.2012.01362.x

**Published:** 2012-06

**Authors:** Yat T Tang, Rong Gao, James J Havranek, Eduardo A Groisman, Ann M Stock, Garland R Marshall

**Affiliations:** 1 Center for Computational Biology, Department of Biochemistry and Molecular Biophysics, Washington University School of MedicineSt. Louis, MO 63110, USA; 2 Howard Hughes Medical Institute, Center for Advanced Biotechnology and Medicine, University of Medicine and Dentistry of New Jersey-Robert Wood Johnson Medical SchoolPiscataway, NJ 08854, USA; 3 Department of Genetics, Washington University School of MedicineSt. Louis, MO 63110, USA; 4 Howard Hughes Medical Institute, Department of Molecular Microbiology, Washington University School of MedicineSt. Louis, MO 63110, USA

**Keywords:** bacterial virulence, docking, drug discovery, gram-negative pathogens, molecular recognition, protein–ligand interactions, protein–protein interactions, response regulators, scoring, structure-based drug design, two-component signal transduction systems, virtual screening

## Abstract

Two-component signal transduction (TCST) is the predominant signaling scheme used in bacteria to sense and respond to environmental changes in order to survive and thrive. A typical TCST system consists of a sensor histidine kinase to detect external signals and an effector response regulator to respond to external changes. In the signaling scheme, the histidine kinase phosphorylates and activates the response regulator, which functions as a transcription factor to modulate gene expression. One promising strategy toward antibacterial development is to target TCST regulatory systems, specifically the response regulators to disrupt the expression of genes important for virulence. In *Salmonella enterica*, the PhoQ/PhoP signal transduction system is used to sense and respond to low magnesium levels and regulates the expression for over 40 genes necessary for growth under these conditions, and more interestingly, genes that are important for virulence. In this study, a hybrid approach coupling computational and experimental methods was applied to identify drug-like compounds to target the PhoP response regulator. A computational approach of structure-based virtual screening combined with a series of biochemical and biophysical assays was used to test the predictability of the computational strategy and to characterize the mode of action of the compounds. Eight compounds from virtual screening inhibit the formation of the PhoP-DNA complex necessary for virulence gene regulation. This investigation served as an initial case study for targeting TCST response regulators to modulate the gene expression of a signal transduction pathway important for bacterial virulence. With the increasing resistance of pathogenic bacteria to current antibiotics, targeting TCST response regulators that control virulence is a viable strategy for the development of antimicrobial therapeutics with novel modes of action.

Infectious diseases have evolved resistance to most clinical antibiotics. Antibiotic resistance occurs at low levels in natural populations, but can become prevalent within a few years of the clinical adoption of an antibiotic. Antimicrobial therapeutics currently in clinical use either inhibit bacterial growth or induce death. One promising strategy is to combat virulence *per se* without inhibiting growth or inducing death, so less selective pressure will cause the bacteria to generate resistance. With the emergence of bacterial strains resistant to multiple antibiotics, there is an urgent need for the development of antibiotics with different modes of action less subjective to the development of resistance.

Two-component signal transduction (TCST) is the predominant signaling scheme in bacteria to sense and respond to environmental changes for survival and proliferation (1–5). TCST regulatory systems are modular in terms of their arrangement of domains within their proteins within various pathways. In general, TCST regulatory systems are comprised of a transmembrane sensor histidine kinase (HK) and an intracellular receiver response regulator (RR) with conserved sequence, structural, and biochemical properties, allowing them to readily adapt to various modes of intracellular signaling. These signaling systems typically couple environmental stimuli to an adaptive response, participating in fundamental processes such as regulating metabolism, as well as more specialized functions such as controlling virulence for the pathogen’s host.

The PhoQ/PhoP two-component regulator system is a major regulator of virulence in *Salmonella enterica* serovar Typhimurium and also in a number of other gram-negative bacterial pathogens (e.g., *Shigella flexneri*, *Yersinia pestis*, *Neisseria meningitidis*) (6–11). PhoQ/PhoP in *S. enterica* is activated by low extracellular Mg^2+^ levels, acidic pH, and antimicrobial peptides (typical of human gut conditions during infection) to control various physiological and virulence functions (7,12–14). In the signaling cascade (Figure 1), the PhoQ histidine kinase is activated by low extracellular magnesium levels and is autophosphorylated at a histidine residue. PhoQ subsequently transfers the phosphate group from the conserved histidine of PhoQ to the conserved aspartate on the PhoP response regulator. Phosphorylation of PhoP presumably induces a conformational change to mediate homodimerization for DNA binding. The PhoP homodimer functions as a transcription factor by recognizing and binding to *phoP* boxes in promoters of PhoP-regulated genes. Through this mechanism, PhoP regulates expression of approximately 3% of the *Salmonella* genes in response to low magnesium levels to control physiological and virulence functions. The *S.* *enterica* PhoQ/PhoP signaling pathway is one of the better characterized bacterial TCST systems demonstrated to be important for virulence regulation.

**Figure 1:**
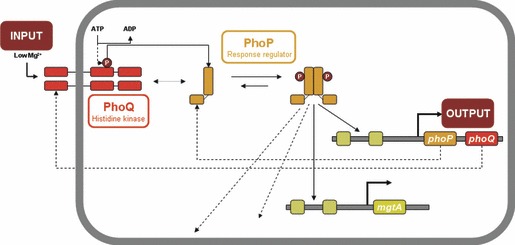
Schematic diagram of the *Salmonella enterica* PhoQ/PhoP two-component signal transduction system. PhoQ is the sensor histidine kinase, and PhoP is the response regulator. PhoQ senses low extracellular magnesium levels, leading to autophosphorylation at a conserved histidine residue. PhoQ transfers to phosphate group to a conserved aspartate residue on PhoP. Phosphorylation of PhoP mediates activation by causing a conformational change, allowing PhoP to homodimerize. PhoP recognizes *phoP* boxes at its DNA promoters (e.g., *phoP*, *phoQ*, *mgtA*) and function as a transcription factor to regulate virulence gene expression (including positive feedback loop).

PhoP is a member of the OmpR/PhoB structural family, the largest structural family making up approximately 30% of all TCST response regulators. OmpR/PhoB family members are typically composed of two domains connected by a flexible linker: an N-terminal receiver domain and a C-terminal DNA-binding domain. Response regulators of the OmpR/PhoB family are characterized by a conserved α4-β5-α5 structural motif of the receiver domain, a plastic surface important for dimerization. Owing to the conservation and importance of the α4-β5-α5 structural motif for function among OmpR/PhoB family members, it was hypothesized that bacterial virulence could be inhibited by disruption of the PhoQ/PhoP signaling pathway, specifically by targeting the α4-β5-α5 interface of the PhoP response regulator. Preventing this essential protein–protein interaction (PPI) would inhibit formation of the PhoP-DNA complex and its function as a transcription factor to regulate gene expression. PhoP was chosen in this study as a prototype of the OmpR/PhoB family of response regulators to probe for PPI ‘hot-spots’ at the α4-β5-α5 interface (Figure 2), in efforts to identify and characterize potential binding sites. The response regulator N-terminal α4-β5-α5 interface shares a common set of hydrophobic and charged residues involved in van der Waals contact and salt-bridges important for homodimerization and function and is conserved among other response regulators across different bacteria species (2,15). High-resolution X-ray crystal structures of both inactivated (Protein Data Bank (PDB) ID: 2PKX) and activated (PDB ID: 2PL1) *Escherichia coli* PhoP were available with an interface highly similar in sequence to the one in *S. enterica* (differing only by one residue at the α4-β5-α5 interface) (16). For these reasons, *S. enterica* PhoP was an attractive target for investigation via structure-based drug design to test the effects of response regulator inhibition and its potential for virulence regulation.

**Figure 2:**
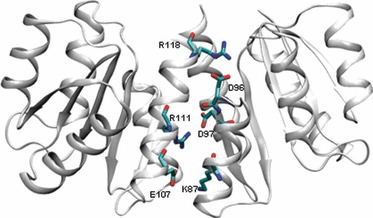
Critical salt-bridges at the PhoP α4-β5-α5 interface important for PhoP homodimerization and function. Residues important for dimerization (site-directed mutagenesis studies, Stock, Gao & Stock unpublished) are shown in capped sticks and labeled by their residue name and number. Mutation of one of these residues decreases its ability to homodimerize. The PhoP homodimer (PDB ID: 2PKX) (cartoon) binds to *phoP* boxes in promoters of PhoP-regulated genes to modulate virulence gene expression.

Targeting bacterial signal transduction systems has only recently been demonstrated to be an effective potential strategy for antibiotics development. Rasko *et al.* (17) targeted the sensor TCST component, the QseC histidine kinase, by the prevention of autophosphorylation, which led to disruption of the signaling cascade important for virulence regulation. Shaknovich *et al.* demonstrated the feasibility of a small molecule for homodimer inhibition and virulence gene regulation when they discovered virstatin to target the *Vibrio cholerae* ToxT (18,19). These studies demonstrated the feasibility of drug-like molecules targeting gene expression important for virulence regulation as a potential strategy for antibiotics development.

A prototype of the predominant class of bacterial signal transduction important for bacterial virulence is investigated as a proof-of-concept study toward this new strategy for antibiotics development. TCST systems predominate in control of bacterial expression and are completely absent in humans, making them an attractive class of targets for the development of new antibiotics with novel modes of action. To our knowledge, there are currently no known inhibitors of TCST response regulators. Drug-like compounds targeting PhoP, specifically the functionally important α4-β5-α5 interface, should selectively disrupt its function as a transcription factor and inhibit the expression of critical virulence genes. In this study, a hybrid approach coupling computational and experimental methods (Figure 3) was used to predict, validate, and characterize drug-like inhibitors of the *S. enterica* PhoP response regulator.

**Figure 3:**
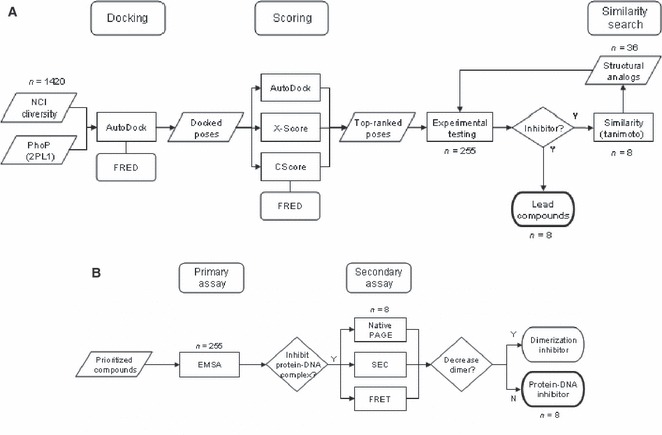
Schematic diagram of the computational (A) and experimental (B) workflow to predict and test for PhoP response regulator inhibitors. In the computational method (A), a drug-like version of the NCI Diversity library (*n* = 1420) was screened for inhibitors of activated PhoP (PDB ID: 2PL1). Docking was performed using AutoDock 4.0.1 and FRED (OpenEye). The predicted binding poses were rescored using X-Score and CScore. A consensus scoring scheme composed of the predict affinities from AutoDock, X-Score, and CScore was used to rank the compounds. The top-ranked compounds were tested experimentally. Similarity search (Tanimoto Index) was performed on eight compounds with inhibition activity to search for structural analogs. In the experimental strategy (B), the set of prioritized compounds were initially tested using EMSA. Compounds displaying inhibition activity by EMSA were further characterized for effects on dimerization using native PAGE, SEC, and FRET. Eight compounds inhibited formation of the protein-DNA complex necessary for virulence gene regulation.

## Methods and Materials

### Overview of computational strategy

Figure 3A illustrates an overview of the computational strategy to prioritize compounds targeting the α4-β5-α5 interface of PhoP. Structure-based virtual screening was used to screen a drug-like version of the National Cancer Institute (NCI) Diversity library (*n* = 1420) (20) with the crystal structure of the activated PhoP (PDB ID: 2PL1) (16). The docking procedure (labeled ‘Docking’) was performed using AutoDock to predict binding poses. A consensus scoring method (labeled ‘Scoring’) consisting of the predicted binding affinities from AutoDock (21), X-Score (22), and CScore^a^ was used to better assess the binding affinities of the resulting poses. The top-ranked compounds were tested experimentally to assess biological activity and characterize the mode of action. As an alternative computational method to identify potential PhoP inhibitors, FRED^b^ was used for both docking and scoring (consensus method using PLP, ChemScore, OEChemScore). The top-ranked compounds from FRED (*n* = 40) were also tested experimentally.

From the experimental results, a similarity search based on the Tanimoto index (labeled ‘Similarity Search’) was performed to identify structural analogs (*n* = 36). These analogs were also experimentally tested. A total of 255 compounds were tested experimentally and verified eight compounds that inhibited formation of the PhoP-DNA complex necessary for gene regulation.

### Compound library and PhoP X-ray crystal structures

A drug-like version of the National Cancer Institute (NCI) Diversity I library composed of 1420 compounds [derived from a larger version of the NCI library of 140 000 compounds filtered based on criteria characteristic of drug-like compounds derived from Lipinski’s Rule of 5 (23)] was screened (20,24). Compounds in the NCI Diversity library have purity 90% or better as characterized by liquid chromatography-mass spectrometry (LC-MS). The compound library was downloaded in SD format (http://gfscore.cnrs-mrs.fr/download/diversity1440.sdf) and converted to mol2 format using Open Babel (http://www.openbabel.org). Inactivated and activated *E. coli* PhoP (PDB ID: 2PKX and 2PL1, respectively) were downloaded from the Protein Data Bank (16).

### Structure-based virtual screening

#### Autodock

AutoDock 4.0.1 was used to predict binding poses for compounds in the NCI Diversity library for experimental testing (21,25). AutoDock was used to convert the ligand structures from the mol2 format to the AutoDock pdbqt format, with explicit hydrogen atoms and calculated Gasteiger charges (26). AutoDock Tools (ADT) was used to prepare the protein structures (27). Polar hydrogens were added to the protein target, and Gasteiger charges were assigned. The structure files were saved in the pdbqt format.

**Figure 4:**
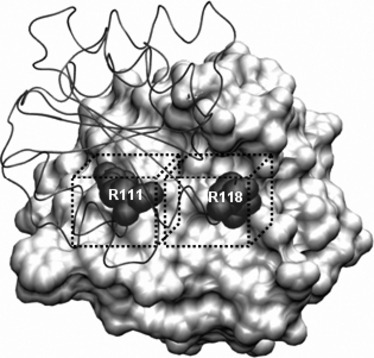
To prioritize compounds in NCI Diversity (*n* = 1420) to identify PhoP dimerization inhibitors, docking searches were constrained at the interface regions where salt-bridges critical for dimerization are located (AutoDock grid boxes used illustrated by hashed lines). Residues critical for dimerization (R111 and R118) are labeled and shown in CPK, in addition to PhoP (PDB ID: 2PKX) chain A (ribbon) and chain B (surface).

Docking was performed to identify low-energy conformations (binding poses) of the compounds to sterically and chemically complement the binding site. The protein search area (grid spacing of 0.375 Å with dimensions of 40 × 40 × 40 Å) was centered at the α4-β5-α5 motif where residues critical for dimerization in PhoP (e.g., Arg 111 and Arg 118 of activated PhoP) and other OmpR/PhoB family members are located, as shown in Figure 4 (28). Lamarckian genetic algorithm was used to perform the ligand conformational searches to result in 30 binding poses for each ligand. The default parameters were used; to increase the sampling and accuracy, the following parameters were modified: ga_pop_size = 200; ga_num_evals = 5 000 000; ga_run = 30.

A drug-like version of the NCI Diversity library (1420 compounds) (20) filtered based on drug-like and lead-like features was screened using AutoDock 4.0.1 (29). Virtual screening was performed on the *E. coli-*activated PhoP (PDB ID: 2PL1), because the sequence at the dimer interface was highly similar to the *S. enterica* PhoP. (*S. enterica* PhoP contains SER 93, while *E. coli* PhoP has ALA 93. Modeling was not performed to change the ALA 93 to SER 93 because this residue was not present in the specified docking regions.) AutoDock was used to generate docked poses of the NCI Diversity compounds at the PhoP dimer interface.

#### FRED (OpenEye)

fred was used as an alternative computational tool for both docking and scoring. OMEGA2 was used to generate bioactive ligand conformations according to the parameters from the study by Bostrom *et al.* (30). Default parameters were used for fred docking searches. Binding site was marked by using a docked ligand from the NCI Diversity library and specifying the regions within 5 Å of it. The default fred scoring function was used to predict binding affinities to rank ligands for experimental testing.

### Rescoring of predicted poses

Owing to the known limitations of scoring functions to accurately predict binding affinities (31–33), especially in the case where the compounds are docked to a novel target with a relatively flat binding site typical of protein dimer interfaces, a consensus scoring scheme was used to prioritize and identify tight-binding ligands (31,33–37). Consensus scoring has proven to be effective in increasing the enrichment rate in a number of studies (37,38). Ligand binding poses predicted by AutoDock were rescored using CScore in sybyl 7.3, which is composed of four separate scoring functions (D-score, ChemScore, PMF-score, and G-score), and X-Score (22,39). The consensus score for CScore resulted in integer scores between 1 and 4, with four being the highest. Docked poses were then sorted based on the consensus CScore value and subsequently by the ChemScore binding affinities. The consensus score estimated by X-Score with default parameters, composed of three different scoring functions (HPScore, HMScore, and HSScore), was used. The top 15% of the docked poses ranked by each of the scoring functions (the AutoDock binding free energy, the CScore consensus method, and the X-Score consensus method) were selected, and the docked poses found in all three scoring methods (6390 poses representing 179 compounds) were selected for experimental testing.

This computational protocol of docking and consensus scoring has been demonstrated to be effective in our research group for identifying lead compounds from the NCI Diversity library for various PPI studies (40). Because there are no known inhibitors of PhoP or any TCST response regulators with which to validate a computational protocol (there are only a small number of structural examples of protein–protein interaction inhibitors available as reviewed by Wells (41) and no examples to our knowledge with a binding site that is structurally related to PhoP), this protocol was initially used for virtual screening.

### Overview of experimental strategy

Figure 3B illustrates an overview of the experimental strategy to assess biological activity and characterize the mode of action of the predicted compounds. As a primary assay, electrophoretic mobility-shift assays (EMSA) were used to assess whether the compounds (*n* = 255) can inhibit the formation of the *S. enterica* PhoP-DNA complex necessary for gene regulation. To test whether the compounds active in EMSA (*n* = 8) affected PhoP dimerization, secondary assays used included native polyacrylamide gel electrophoresis (PAGE), size-exclusion chromatography, and Forster resonance energy transfer (FRET)-based assay. Native PAGE was used to detect qualitative effects on *S. enterica* PhoP dimerization. SEC was used to better characterize the compounds’ ability to inhibit *S. enterica* PhoP dimerization by measuring the monomer–dimer profile. FRET was used as a higher-throughput method to quantify the effects of dimerization on *E. coli* OmpR/PhoB members to assess compound selectivity at the α4-β5-α5 interface. Experimental results from the primary and secondary assays suggest the compounds inhibit formation of the PhoP-DNA complex, not by dimer inhibition, but in an allosteric manner to prevent DNA binding or by blocking the C-terminal DNA-binding domain. Potential mode of actions will be presented in a greater detail in Results and Discussions.

### Electrophoretic mobility-shift assays

As a primary assay, an electrophoretic mobility-shift assay, also known as a gel-shift assay, was used to test the ability of the compounds to inhibit formation of the PhoP dimer-DNA complex necessary for gene expression. In the gel-shift assays, the protein is first activated by phosphorylation to induce the monomer–dimer equilibrium. Radiolabeled DNA is incubated with the protein, and the compound of interest is subsequently added to the mixture. After an incubation period, the sample is loaded and electrophoresed on a gel to observe formation of the protein-DNA complex. Compounds that inhibit protein-DNA complex formation will lead to a darker band further down in the gel representing unbound DNA, while compounds that do not effect complex formation will result in a single band higher up on the gel (bound complex). Band intensities can be quantified and fitted to a sigmoidal curve to estimate IC_50_ values in dose–response experiments.

PhoP was activated by *in vitro* phosphorylation with acetyl phosphate to induce the formation of monomer–dimer equilibrium. Compounds dissolved in DMSO (99.9%) were then incubated with the monomer–dimer mix, which did not exceed 1% volume of DMSO in the final solution concentration. Radioactive-labeled DNA containing *phoP* boxes (GGTTTAxxxxTGTTTA) were subsequently incubated with the mix to allow formation of the PhoP-DNA complex. Samples were loaded on 4–20% Tris-borate-EDTA (TBE) gels (Invitrogen) and electrophoresed. Gels were dried and analyzed using a phosphorimager (FujiFilm BAS-5000). Compounds that did not affect DNA binding displayed a single band upstream that represented the bound PhoP-DNA complex. Compounds that inhibited formation of the PhoP-DNA complex displayed a ‘shift,’ resulting in a band of the PhoP-DNA complex and/or a downstream band that represented unbound DNA.

ImageJ^c^ was used to quantitate the EMSA band intensities (for both bands representing bound and unbound DNA). KaleidaGraph^d^ was used to perform the curve fitting using a sigmoidal function to derive the IC_50_ values from dose–response experiments.

The *phop-*DNA fragments for electrophorectic mobility-shift assays were amplified by PCR using primers 312 and 369, and genomic DNA of wild-type *S. enterica* as template. The DNA fragments were isolated by running an electrophoretic gel and purified using QIAquick columns (Qiagen). To radiolabel the DNA fragments, 100 ng of phop-DNA was used with T4 polynucleotide kinase and γ-^32^P ATP and incubated at 37 °C for 1.5 h. Unincorporated DNA was removed using G-50 microcolumns (Amersham). 20 000 CPM of labeled probe (∼12 fmol), 200 ng poly(dI-dC)-poly(dI-dC) (Amersham), and phosphorylated PhoP-His_6_ were mixed with binding buffer (50 mm Tris-HCl pH 8.0, 50 mm KCl, 50 μg/mL BSA) in a total volume of 20 μL and incubated for 20 min at room temperature.

For electrophoretic mobility-shift assays, *S. enterica* PhoP-His_6_ was phosphorylated with acetyl phosphate. PhoP (0.6–1.2 nmol) was incubated in 50 μL of phosphorylation buffer (50 mm Tris-HCl pH 7.5, 50 mm KCl, 10 mm MgCl_2_) containing 10 mm acetyl phosphate (Sigma-Aldrich) for 2.5 h at room temperature. Excess acetyl phosphate was removed from phosphorylated PhoP-His_6_ using a Micro Bio-Spin 6 Chromatography Column (Bio-Rad, Hercules, CA, USA) equilibrated with Tris buffer. Phosphorylated PhoP-His_6_ was kept at 4 °C and used within 24 h.

### Similarity search

To identify similar compounds of the experimentally confirmed inhibitors, a similarity search (Tanimoto index) using the NCI website (http://129.43.27.140/ncidb2/) was performed to search the larger NCI library of 140 000 compounds. Using the experimentally confirmed compounds as the query, 36 compounds (Supporting Information) from the full NCI library were found, ordered, and experimentally tested by EMSA for inhibition activity.

### Native PAGE

To test the effect of compounds on PhoP dimerization, a native polyacrylamide gel electrophoresis (PAGE) assay was used. The protein is first activated by phosphorylation to induce formation of the monomer–dimer equilibrium (same method as described in EMSA). The compound of interest is incubated with the equilibrium mixture. Samples are loaded and electrophoresed on a native PAGE gel to separate the monomer and dimer states. Proteins can be visualized by gel staining. Compounds with no effects on dimerization should display two bands: one less intense and more downstream band representing the monomer, and a darker and more upstream band representing the dimer. Compounds with inhibition effects on dimerization will display a more intense band representing the monomer and a less intense band representing the dimer.

*Salmonella enterica* PhoP-His_6_ was phosphorylated in the same manner as by EMSA. Compounds were incubated with the active protein for 30 min, and loading buffer was added after. Samples were loaded and ran on a native gel (6% Tris–glycine; Invitrogen, Grand Island, NY, USA) at 4 °C. Gels were then stained with coomassie blue to visualize the bands representing the protein.

### Size-exclusion chromatography (SEC)

Size-exclusion chromatography was used to better characterize the monomer–dimer distribution. Protein is activated by phosphorylation (similar manner to EMSA and native PAGE) to induce the monomer–dimer equilibrium. Compound of interest is incubated with the equilibrium mix and injected to and separated by the size-exclusion column. Relative absorbance (A_280_) is detected from the elutions to quantify the amount of protein present. Compounds that do not inhibit dimerization will display a larger peak in the earlier elution representing the dimer, and a smaller peak in a later elution representing the monomer. Compounds that do not effect dimerization will not display any changes in the monomer–dimer absorbance profile. Purified PhoP-His_6_ was phosphorylated using 50 mm ammonium phosphoramidate [synthesized by the method of Sheridan *et al.* (42)] and 20 mm MgCl_2_ for 30 min at room temperature. Compounds were subsequently incubated with PhoP for 30 min. Samples of inactivated and activated PhoP (100 μL) were individually applied to a Superdex 75 column (GE Healthcare, Piscataway, NJ, USA) equilibrated with elution buffer (50 mm Tris/HCl, pH 8.0, 150 mm KCl). Proteins were eluted in the same buffer at a flow rate of 0.5 mL/min. Protein concentration was assessed by measuring the OD_280_. Fractions were collected and analyzed using native PAGE. A molecular weight standards kit (Sigma-Aldrich, St. Louis, MO, USA) was applied to the column for calibration.

### Forster resonance energy transfer analyses

Forster resonance energy transfer (FRET) analyses were used to detect and quantify effects on dimerization on *E. coli* PhoP. In FRET, a cyan fluorescent protein-fused PhoP (CFP-PhoP) and a yellow fluorescent protein-fused PhoP (YFP-PhoP) are activated by phosphorylation. Phosphorylation induces dimerization between CFP-PhoP and YFP-PhoP and brings the CFP and YFP in proximity where an energy exchange occurs. PhoP dimerization is characterized by a decrease in cyan emission and an increase in yellow emission. The rate of FRET increase depends on the rates of phosphorylation and dimerization. Compounds that inhibit dimerization will lead to a decrease in the FRET signal, while compounds that do not effect dimerization will not change the FRET signal.

Forster resonance energy transfer analyses of fluorescent protein (FP) fused PhoP proteins, CFP-PhoP and YFP-PhoP, were performed as described in Gao *et al.* (43). Phosphorylation of FP-PhoP was initiated by addition of MgSO_4_ and phosphoramidate to the mixture of CFP-PhoP, YFP-PhoP, and indicated compounds. The final concentrations were 0.6 μm CFP-PhoP, 2.5 μm YFP-PhoP, 20 mm phosphoramidate, 5 mm MgSO_4_, 100 μm compounds, and 1% (v/v) DMSO. Fluorescence was followed at 475 and 530 nm with excitation at 430 nm. The ratio of 475 and 530 nm emissions was defined as the FRET ratio to monitor the interaction between CFP-PhoP and YFP-PhoP.

### *Salmonella enterica* PhoP expression and purification

*Salmonella enterica* PhoP response regulator with a C-terminal His_6_ tag was overexpressed in *E. coli* strain BL21(DE3) transformed with the pT-7-7 plasmid. Cells were grown in Luria-Bertani medium with ampicillin (100 mg/L) and incubated until mid-logarithmic phase at 37 °C. Overexpression was induced with 1 mm isopropyl-β-D-thiogalactopyranoside (IPTG) and incubated overnight at 25 °C.

For purification, cells were harvested by centrifugation and then washed and resuspended in PBS, and stored overnight in −80 °C. Cells were then thawed at 4 °C, suspended in lysis buffer (50 mm NaH_2_PO_4_, pH 8.0, 300 mm NaCl, 10 mm imidazole) and lysed by sonication. Cells were then centrifuged and the cell lysate was collected as the supernatant. Cell lysate was applied to a Ni^2+^ column (GE Healthcare). Unbound proteins were removed with elution with Buffer A (50 mm Tris–HCl pH 8.0, 500 mm NaCl, 10 mm imidazole). Bound proteins were eluted with a 0–100% gradient of Buffer A to Buffer B (50 mm Tris–HCl pH 8.0, 100 mm NaCl, 500 mm imidazole). Fractions were collected and analyzed by SDS/PAGE. Proteins were concentrated using Amicon Ultra-15 filters (Millipore) and stored in storage buffer (20 mm Tris pH 7.8, 100 mm KCl, 20% glycerol) at −80 °C. Protein concentrations were determined by measuring the OD_280_ (NanoDrop spectrophotometer). Purity was assessed by SDS/PAGE.

## Results and Discussion

### Computational strategy identified inhibitors that potentially bind at the PhoP α4-β5-α5 interface

A total of 259 compounds were tested experimentally. From the results obtained by AutoDock and the consensus scoring scheme, the following number of compounds include 119 compounds that used a search grid centered at Arg 111 and 60 compounds that used a search grid centered at Arg 118. From the results obtained by docking using FRED (search area centered at Arg 111), 40 compounds were tested.

### Eight compounds inhibited formation of the PhoP-DNA complex

**Figure 5:**
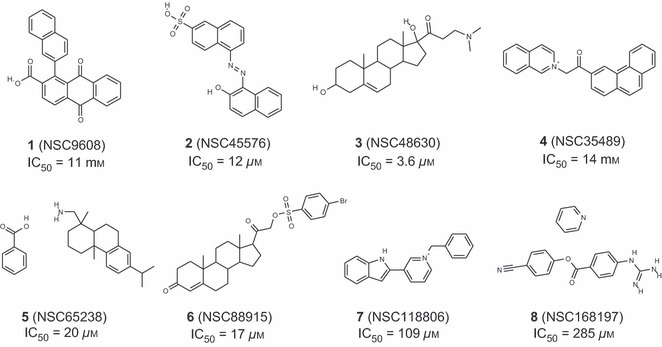
Chemical structures of the eight compounds (**1**-**8**) that inhibited formation of the PhoP-DNA complex by electrophoretic mobility-shift assays (EMSA) with their NSC number and their estimated IC50 values.

**Figure 6:**
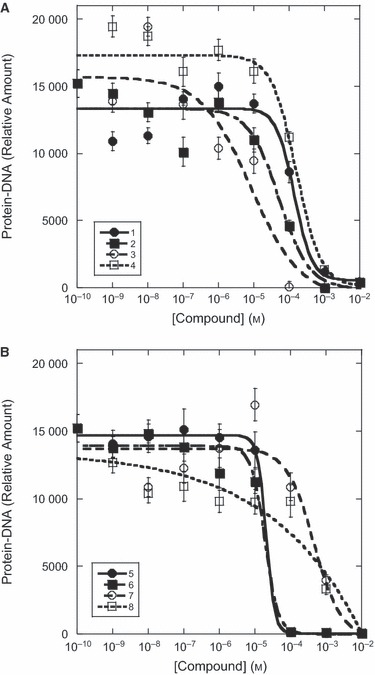
Dose response curves (fitted by sigmoidal function, KaleidaGraph) from electrophoretic mobility-shift assays in presence of compounds **1**-**4** (A) and **5**-**8** (B). The points plotted represent the relative amount of PhoP-DNA complex formed in EMSA experiments.

A total of 255 compounds were tested by EMSA: 119 compounds from the Arg 111 binding site screen using AutoDock, 40 compounds from the Arg 111 binding site screen using FRED, 60 compounds from the Arg 118 binding site screen, and 40 compounds identified from a Tanimoto similiarity search for analogs of the initial hits within the entire NCI library. Eight compounds (NSC9608, NSC45576, NSC48630, NSC35489, NSC65238, NSC88915, NSC118806, and NSC168197) (Figure 5) displayed inhibition activity via disruption of the PhoP-DNA complex. The eight compounds inhibited PhoP-DNA complex formation in a dose-dependent manner (Figure 6), with six of the eight compounds displaying an IC_50_ in the micromolar range (from 3.6 to 285 μm).

Analogs (36 in total) of the eight inhibitors based on Tanimoto similarity (90% similar) from the NCI library (140 000 compounds) did not display inhibition activity by EMSA. Inactivity of these analogs may be due to the sensitive preference of the PhoP binding site at the α4-β5-α5 interface.

### Compounds did not effect PhoP dimerization

**Figure 7:**
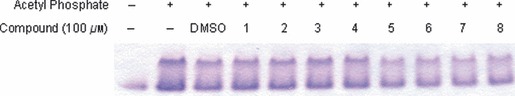
Native PAGE results of S. enterica PhoP in presence of eight inhibitor compounds (**1**–**8**). PhoP dimerization was induced by phosphorylation via acetyl phosphate. An unphosphorylated sample without compound (first lane from left) was used as a negative control. Samples with buffer only (second lane from left) and with 1% (v/v) DMSO (third lane from left) were used as positive controls.

The eight compounds did not display any effects on band intensity for the band representing the PhoP monomer and dimer in the native PAGE assay (Figure 7). These results suggested that the compounds do not act as dimerization inhibitors. However, band intensity changes may not have been observed because of the inability of the gel to distinctly separate the phosphorylated monomer and dimer species.

The eight compounds also did not display any changes on the monomer–dimer profile of PhoP as compared with a control (DMSO) using size-exclusion chromatography, which performs a better separation and characterization of the oligomeric states than native PAGE. One caveat of using SEC as a binding assay is that a compound exhibiting a rapid off-rate cannot be detected with SEC because of time (30 min.) needed to perform the separations.

**Figure 8:**
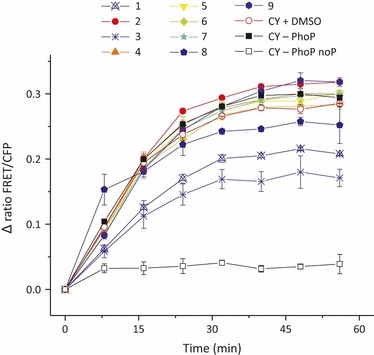
Time-dependent FRET of *Escherichia coli* FP-PhoP pairs in the presence of inhibitor compounds (**1**–**8**). Inactive PhoP (CY-PhoP no P) was used as a negative control, while activated PhoP (CY-PhoP) and sample with DMSO (CY +DMSO) were used as positive controls.

Results from the FRET assays using *E. coli* PhoP also did not suggest any degree of dimer inhibition (Figure 8). However, with the use of CFP-PhoP and YFP-PhoP, one possibility is that the compounds may bind to CFP or YFP instead of PhoP, resulting in undetected inhibition effects. Some compounds (e.g., **1**, **2**) were inherently colored and affected the detection of FRET signal and, therefore, could not be properly tested for dimer inhibition. Compounds **1**, **3**, and **8** displayed a smaller FRET ratio changes as compared with the positive control sample (e.g., CY +DMSO, CY PhoP). However, compounds **1** and **3** interfere with the FP fluorescence, while compound **8** alters the fluorescence ratio of FP-PhoP pairs. Therefore, the FRET method is not sufficient to assess the inhibition of compounds **1**, **3**, and **8**. FRET also cannot detect compounds that bind to the α4-β5-α5 interface to inhibit DNA binding in an allosteric manner, because only the N-terminal of the PhoP response regulator bound to CFP or YFP was used.

### Compounds suggested selective inhibition for *Salmonella enterica* PhoP

The 8 compounds did not display any effects on the signal in the FRET assays with *E. coli* PhoP, which suggested no inhibition of dimerization (Figure 8). The compounds may bind to the *E. coli* PhoP α4-β5-α5 interface, but have no effects on dimerization. However, because the *E. coli* PhoP linker and C-terminal were not included in the FRET analyses, there remains a possibility that the compounds may bind to *E. coli* PhoP to inhibit DNA binding. These results obtained so far suggested that the compounds might selectively bind to the *S. enterica* PhoP α4-β5-α5 dimer interface to inhibit formation of the protein-DNA complex, perhaps by an allosteric mechanism.

### Inhibitors may act by a different mode of action

Experimental results from a series of *in vitro* and biophysical assays (EMSA, Native PAGE, SEC, FRET) suggested that the compounds tested disrupt PhoP-DNA complex formation, but not via direct homodimer inhibition. Instead, one potential mode of action is to bind to the C-terminal DNA-binding domain, a more direct mechanism to disrupt formation of the PhoP-DNA complex. Another possible mode of action is by binding to the N-terminal regulatory receiver domain or the linker region to act in an allosteric manner and prevent conformational changes necessary for DNA binding. The former may prove to be a more effective strategy for selective inhibition of response regulators, because of the conserved α4-β5-α5 structural motif of the dimer interface.

Virtual screening may have helped to identify compounds binding the interface. The inhibitors did not block dimerization, but did prevent formation of PhoP-DNA complex necessary for gene regulation, perhaps by binding at the α4-β5-α5 interface and acting by an allosteric mechanism.

### Challenges of structure-based design to target protein–protein interactions

In the pursuit of discovering PPI inhibitors, the original objective was to use small molecules as probes to identify and characterize potential binding sites at the α4-β5-α5 interface of *S. enterica* PhoP to block homodimerization. Through the discovery of dimerization inhibitors presumably binding at the sites where the critical residues are located, it was hoped that the findings would lead to a better understanding of the physical properties underlying molecular recognition of protein–ligand interactions at PPI. While the original intent of this study was to target the α4-β5-α5 interface to block dimerization, the experimental results suggest an unexpected finding: drug-like compounds may bind at the α4-β5-α5 interface and function in an allosteric manner and cause a conformational change to prevent DNA binding. Further experimental characterization (e.g., crystallography, NMR) will be necessary to identify potential binding sites and to elucidate the atomic details of the complexes to determine the mode of action.

Structure-based discovery of protein–protein interaction inhibitors remains a significant challenge as seen by the high percentage of false positives from the computational predictions in this study. Docking and structure-based design methods to incorporate protein flexibility will need to be used to identify and design potential allosteric inhibitors or ones that bind by an induced-fit mechanism. Another potential limitation is in the scoring functions used for virtual screening, which cannot accurately predict binding affinities, especially for systems that are not present in the scoring function training sets (e.g., binding sites with relatively flat surfaces). Even if the docking modes may be correctly identified, limitations in scoring accuracy may classify potential tight-binding ligands as weak-binders, resulting in a high percentage of false positives. Perhaps, the use of first-principle methods for estimating binding affinities may help circumvent this limitation, assuming that the correct ligand conformation was predicted by the docking procedure.

Also, availability of a high-resolution crystal structure of PhoP bound to its DNA promoter should better elucidate the conformation of the α4-β5-α5 interface in the biologically active form bound to DNA and provide atomic-level detail of potential binding sites necessary for docking and structure-based drug design. However, inherent inaccuracy of using a ‘static’ structure for molecular design may lead to false negatives using structure-based design methods without incorporation of protein flexibility. For more accurate computational modeling, in particular to target PPI, improvements in both docking and scoring methods are necessary. With the increasing interest in targeting PPI and availability of structural and binding affinity data, development of more accurate and robust SBDD methods to target PPI will become possible.

### Discovery of first-in-class two-component signal transduction response regulator inhibitors

In this study, eight first-in-class inhibitors of the *S. enterica* PhoP TCST response regulator were discovered using a hybrid approach coupling computational and experimental methods for molecular design. Potential mode of action of the compounds was characterized by a series of *in vitro* and biophysical assays. Compounds may potentially bind at the α4-β5-α5 interface and act as allosteric inhibitors, rather than dimerization inhibitors, to prevent DNA binding. Discovery of first-in-class PhoP inhibitors should serve as a proof-of-concept for targeting TCST response regulators as a novel strategy to inhibit bacterial virulence.

## Conclusions and Future Directions

Targeting two-component signal transduction response regulators to modulate virulence gene expression is a promising strategy for antibiotics development. With the increasing resistance of bacterial pathogens to current therapeutics, antibiotics that can prevent virulence instead of inhibiting growth or inducing death may lead to less selective pressure for the generation of resistance.

In this study, eight compounds have been discovered by coupling computational and experimental methods to disrupt formation of the *S. enterica* PhoP-DNA complex necessary for gene regulation. Eight compounds inhibited the PhoP-DNA complex formation in a dose-dependent manner by EMSA. Based on the experimental results, the PhoP inhibitors may potentially bind to the plastic α4-β5-α5 interface and act by an allosteric mechanism to prevent DNA binding. Alternative modes of action include binding to other regions of the N-terminal domain to act in an allosteric manner or the C-terminal DNA-binding domain to directly inhibit formation of the PhoP-DNA complex. Experimental results obtained from a series of *in vitro* and biophysical assays suggest a potential of these compounds to inhibit bacterial virulence.

Further elucidation of the mode of action to assess the potential of the eight compounds as virulence inhibitors is planned. Structural analogs of the eight compounds can be designed to enhance affinity and characterize structure-activity relationships. Structural studies such as X-ray crystallography and NMR must be performed to validate the mode of action and elucidate the protein–ligand interactions in atomic detail.

## Footnotes

a CScore (2005). *CScore*. Saint Louis, MO: Tripos, Inc.

b FRED (2009). *FRED*. Sante Fe, NM: OpenEye Scientific Software.

c ImageJ (2008). *ImageJ*. Bethesda, MD: National Institutes of Health.

d KaleidaGraph (2008). KaleidaGraph. Reading, PA: Synergy Software.

## Abbreviations

TCST: two-component signal transduction
RR: response regulators
SBDD: structure-based drug design
PLI: protein–ligand interactions
PPI: protein–protein interactions
EMSA: electrophoretic mobility-shift assays
SEC: size-exclusion chromatography
FRET: Forster resonance energy transfer
